# Studying humane endpoints in a rat model of mammary carcinogenesis

**DOI:** 10.22038/ijbms.2019.33331.7957

**Published:** 2019-06

**Authors:** Ana I Faustino-Rocha, Mário Ginja, Rita Ferreira, Paula A Oliveira

**Affiliations:** 1 Faculty of Veterinary Medicine, Lusophone University of Humanities and Technologies, Lisbon, Portugal; 2 Center for the Research and Technology of Agro-Environmental and Biological Sciences (CITAB), University of Trás-os-Montes and Alto Douro (UTAD), Vila Real, Portugal; 3 Department of Veterinary Sciences, UTAD, Vila Real, Portugal; 4 Organic Chemistry, Natural Products and Foodstuffs (QOPNA), Mass Spectrometry Center, Department of Chemistry, University of Aveiro, Aveiro, Portugal

**Keywords:** Chemically-induced Humane endpoints Mammary cancer, N-methyl-N-nitrosourea, Rat, Welfare

## Abstract

**Objective(s):**

The present work intended to clearly define the most adequate humane endpoints in an experimental assay of mammary carcinogenesis in rats.

**Materials and Methods:**

Animals were observed twice a day; all parameters were registered once a week and the euthanasia endpoints were established in order to monitor the animal welfare/distress during an experimental assay of chemically-induced mammary carcinogenesis in female rats.

**Results:**

Fourteen animals developed at least one mammary tumor with a diameter >35 mm. No animals exhibited alterations in the remaining parameters that implied their early sacrifice. Statistically significant changes were not observed in the quantitative parameters like the hematocrit and urine specific gravity among groups, not being valuable for the assessment of the health status of animals included in an assay of mammary carcinogenesis for 18 weeks. The remaining humane endpoints seemed to be helpful to monitor the animals’ health status.

**Conclusion:**

The alteration in only one humane endpoint (mammary tumor dimensions) does not imply the animals’ sacrifice; the endpoints should be evaluated in conjunction, in order to define the most adequate time in which the animals should be sacrificed.

## Introduction

Studies in live organisms are essential for a whole understanding of complex diseases, and to search for new and more effective ways to diagnose and treat them (1). Although *in vitro *assays are the first step to study diseases, the *in vivo *experiments constitute an essential link between the *in vitro *studies and clinical trials in humans (2). Indeed, animals have long been used in research protocols aiming to study distinct human diseases, like diabetes (3, 4), cardiovascular diseases (5, 6), Alzheimer (7, 8), obesity (9, 10), cerebral palsy-like (11, 12) and cancer (13–20). 

Mice and rats are the most frequently used animals in experimental protocols performed in the European Union as they have several advantages when compared with other animals, such as their small size, and well-known anatomy, physiology, biochemistry and genetic (21, 22). 

Experimental assays using animals should be performed only after exhaustive studies on cell lines and under the highest standards on animal welfare (1, 2). The term “animal welfare” is commonly used to define the animals’ quality of life, and it includes five freedoms established by UK Farm Animal Welfare (23, 24). In 1959, William Russell and Rex Burch published a book entitled “The Principles of Humane Experimental Technique” where they presented the concept of 3Rs (replacement, reduction and refinement) in an attempt to improve the animal welfare in experimental assays, and therefore improve the quality of biomedical research (25, 26). Beyond the fact that pain and distress inflicted to laboratory animals are ethically unacceptable, they also constitute a potential source of errors in the results of the experiment as they change several physiological parameters, namely serum or plasma concentrations of corticosterone, glucose, growth hormone or prolactin, heart rate or blood pressure (27).

Once the concern about the animal welfare was increasing not only among the researchers, but also in public requiring the minimizing of animal pain and distress, the point at which an animal should be removed from the experimental protocol (defined as humane endpoint) should be defined in the design phase of the study (23). The Canadian Council on Animal Care (CCAC) prepared a list of guidelines for selecting adequate endpoints able to minimize the animal pain and distress without compromising the purposes of the research protocols (28). Additionally to general guidelines, the CCAC also proposed specific guidelines for animal models of cancer research, based on the macroscopic appearance and dimensions/weight of tumors (28). According to CCAC, a system of score sheet should be prepared based on pilot studies in order to measure the alterations in clinical signs (29). Moreover, other institutions, like University of Pennsylvania Institutional Animal Care and Use Committee (IACUC) and United Kingdom Coordinating Committee on Cancer Research employed efforts in order to define humane endpoints for rodent cancer models (28). Despite this, the studies on this field are still scarce and this is an issue in constant evolution.

In this way, considering the experience of our team in this field, the present work intended to clearly define the most adequate humane endpoints in an experimental assay of mammary carcinogenesis in rats. 

## Materials and Methods

***Animals***

Thirty-four female Sprague-Dawley rats of four weeks of age and a mean body weight of 179.45±6.04 g were obtained from Harlan Interfauna (Barcelona, Spain) for a research protocol aiming to evaluate the role of the antihistamine and mast cell stabilizer drug ketotifen in a rat model of chemically-induced mammary carcinogenesis. Animals from MNU-exposed groups (groups I, II and III) were housed in groups of five animals; while animals from ketotifen (group IV) and control (group V) groups were housed in groups of two animals. Animals were housed in the facilities of the University of Trás-os-Montes and Alto Douro (UTAD) under controlled conditions of temperature (23±2 ^°^C), humidity (50±10%), air system filtration (10-20 ventilations/hour) and on a 12 hr:12 hr light:dark cycle. A standard laboratory diet (4RF21^®^, Mucedola, Italy) was supplied throughout the study. All experiments followed the European (Directive 2010/63/EU) and National (Decree-Law 113/2013) legislation on the protection of animals used for scientific purposes. The procedures were approved by the Portuguese Ethics Committee (Approval no. 008961) and Ethics Committee of UTAD (CE_12-2013).

***Experimental protocol***

After one week of quarantine and two weeks of acclimatization to the lab conditions, animals were randomly divided into five experimental groups: group I (MNU; n=10), group II (MNU+ketotifen-1; n=10), group III (MNU+ketotifen-2; n=10), group IV (ketotifen; n=2) and group V (control; n=2). At seven weeks of age, animals from groups I, II and III were intraperitoneally injected with the carcinogen agent *N*-methyl-*N*-nitrosourea (MNU) (Isopac^®^, Sigma Chemical Co, Madrid, Spain) at a dose of 50 mg/kg (volume ranging from 0.72 to 0.92 ml, depending on individual body weight) (29). Animals from groups IV and V were injected with the vehicle (saline solution 0.9%). The administration of the carcinogen was defined as the first day of the experiment. On the day after the MNU administration, animals from groups II and IV received the mast cell stabilizer drug ketotifen (Zaditen^®^, Defiante Farmacêutica SA, Portugal) in drinking water, at a concentration of 1 mg/kg/day, 7 days/week for 18 weeks. Each animal from group III only received the ketotifen after the development of the first mammary tumor. These animals received water until the development of the first mammary tumor. Animals from groups I and V received water throughout the study. 

***Animals’ health status***

A list of biological parameters to be evaluated during the experiment was elaborated prior the study, including: body condition, body weight, food and water intake, posture, coat and grooming, mucosal, eyes, ears and whiskers, mental status, response to external stimuli, hydration status, respiratory rate, heart rate and body temperature. A score from 0 to 3 was attributed for each parameter. Severe alteration in some of these parameters, such as weight loss >20%, severe anemia, moribund or comatous mental status, development of mammary tumors that interfere with animal bodily functions (eat or drink), tumors in contact with cage floor or tumor burden >10% of the animal body weight (>35 mm in a 250 g rat) were considered indicators of animal sacrifice (Table 1) (28–34). 

**Figure 1:**
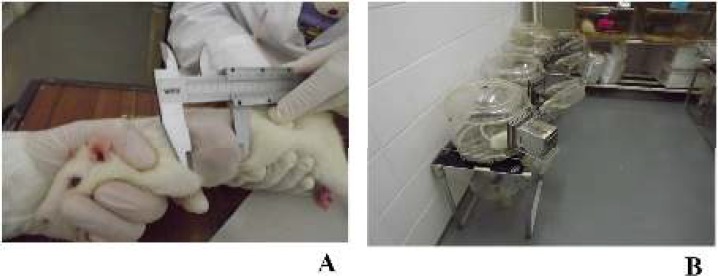
Measurement of a mammary tumor using a caliper (A) and urine sample collection in metabolic cage (B)

During the study, the animals were observed twice a day by the same researchers. Body condition, posture, coat and grooming, mucosal, eyes, ears and whiskers, and mental status were evaluated through animals’ observation. Animals were individually weighed; food and water intake were also determined using a top-loading scale (Mettler PM4000, LabWrench, Midland, ON, Canada). At the end of the experiment, accurate body weight was determined by the subtraction of tumor weight to the animal body weight. Ponderal weight gain was determined as previously defined by Faustino-Rocha *et al.* (30). The response to external stimuli was assessed by evaluating the response of animals to the hand clapping above the cage. Hydration status was evaluated by the skin pinch. Respiratory rate and heart rate were determined by counting the breaths and beats *per *minute, respectively. The body temperature was measured using a laser thermometer. The hematocrit and urine specific gravity were evaluated in different time points throughout the experiment. Mammary chains of all animals were palpated to detect the mammary tumor development and the mammary tumors were measured using a vernier caliper (Vito, Central Lobão SA, Santa Maria da Feira, Portugal) (Figure 1A). The location, macroscopic appearance and burden of mammary tumors were also evaluated (Table 1). 

***Sample collection***

Blood and urine samples were collected in different time points during the experiment to monitor animals’ response to cancer development. Blood samples were collected from tail vein directly into capillary tubes (Haematokrit-Kapillaren, Hirschmann Laborgerte, Eberstadt, Germany) at the 7^th^, 11^th^¸ 16^th^ week of the protocol. At the end of the experiment, during the sacrifice (18^th ^week of the experiment), blood samples were collected from the heart into a syringe, and then the capillary tubes were filled. The tubes were centrifuged (9000 rpm, 5 min, Hermle Z320, Wehingen, Germany) and the microhematocrit was immediately determined. Urine samples were collected using metabolic cages (Tecniplast, Buguggiate VA, Italy) at the 5^th^, 8^th^, 13^th^ and 18^th^ of the experiment. They were centrifuged (3000 rpm, 15 min, Heraeus Labofuge 400R, Thermo Scientific, Waltham, MA, USA) and the urine specific gravity was determined using a refractometer (Atago Co, Tokyo, Japan) (Figure 1B).

***Animals’ sacrifice***

All survived animals were humanely sacrificed at the 18^th^ week of the experiment by an intraperitoneal injection of ketamine (75 mg/kg, Imalgene 1000, Merial SAS, Lyon, France) and xylazine (10 mg/kg, Rompun 2%, Bayer Healthcare SA, Kiel, Germany), followed by exsanguination by cardiac puncture as indicated by the Federation for Laboratory Animal Science Associations (32). Animals were skinned, and the skin was carefully observed under a light to detect the presence of small mammary tumors not previously detected by palpation. All mammary tumors were excised and immersed in buffered formalin for 24 hr for posterior histopathological analysis.

***Statistical analysis***

Data were analyzed with Statistical Package for the Social Sciences (SPSS^®^, version 23 for Windows, SPSS Inc, Chicago, IL, USA). The Shapiro-Wilk test was used to test the normality of the data. Data were analyzed using analysis of variance (ANOVA) with the Bonferroni correction for multiple comparison. All data were expressed as mean±standard error (SE), *P-*values lower than 0.05 were considered statistically significant. 

## Results

***General findings***

No changes in the studied biological parameters that implied the premature sacrifice of any animal were observed during the experiment. One animal from group II (MNU+ketotifen-1) died unexpectedly in the penultimate week of the experiment (17 weeks after MNU administration; mortality index of 10% in this experimental group). Only the animals from the MNU-exposed groups that developed mammary tumors were included in the study (6 animals from group I, 8 animals from group II and 7 animals from group III).

**Figure 2:**
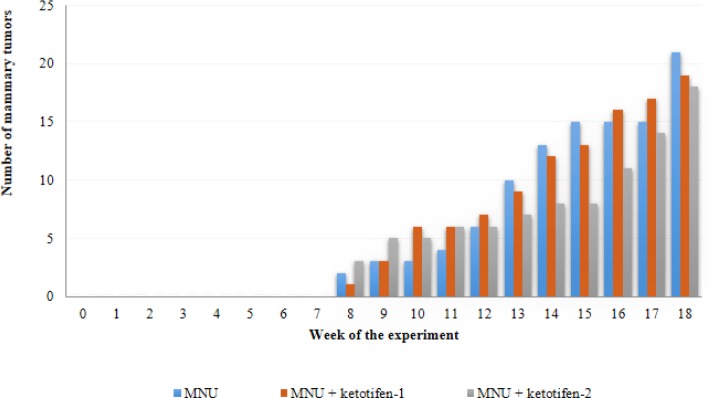
Cumulative number of mammary tumors developed by animals from N-methyl-N-nitrosourea-exposed groups during the experimental protocol

**Figure 3:**
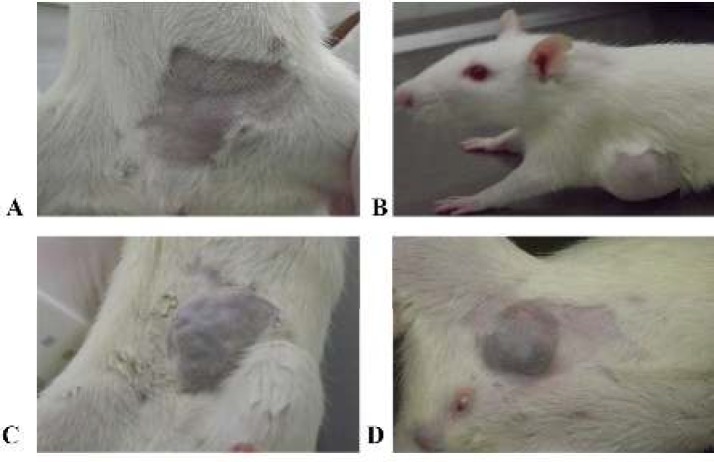
Macroscopic appearance of mammary tumors developed by animals N-methyl-N-nitrosourea-exposed at 15^th^ week of the experimental protocol

**Table 1:** Biological parameters evaluated during the experiment of* N*-methyl-*N*-nitrosourea-induced mammary cancer. A score from 0 to 3 was attributed for each of parameter. A severe alteration in some biological parameters was indication for euthanasia

**Score**		**Parameters**
**General appearance and mental status**	**Body condition**	
	0	Well-conditioned
	1	Under conditioned
	2	Emaciated **(Euthanasia)**
	**Body weight**	
	0	Normal
	1	Weight loss < 10 %
	2	Weight loss 10-20 %
	3	Weight loss > 20 % **(Euthanasia)**
	**Food intake**	
	0	Normal (≈ 5-10 g/100 g of body weight)
	1	Decreased (< 5 g/100 g of body weight)
	**Water intake**	
	0	Normal (≈ 10-15 mL/100 g of body weight)
	1	Decreased (<10 mL/100 g of body weight)
	**Posture**	
	0	Normal position
	1	Changed position (eg. orthopneic position)
	**Coat and grooming**	
	0	Normal
	1	Lack of grooming
	2	Rough coat, chromodachryorrhea
	3	Very rough coat, piloerection, severe chromodachryorrhea
	**Mucosal**	
	0	Normal
	1	Mild anemic
	2	Moderate anemic
	3	Severe anemic **(Euthanasia)**
	**Eyes, ears and whiskers**	
	0	Normal
	1	Partial closed eye, droopy ears, forward whiskers
	2	Complete closed eye, droopy and curved ears, forward and bunched whiskers
	**Mental status**	
	0	Normal (alert, curious, eyes bright)
	1	Lethargic
	2	Stupor
	3	Moribund / Coma **(Euthanasia)**
**Behavior**	**Response to external stimuli (hand clapping above the cage)**	
	0	Normal
	1	Mild response
	2	Moderate response with vocalization
	3	Violent response
**Clinical signs**	**Hydration status**	
	0	Normal (< 2 sec.)
	1	Abnormal skin pinch test (> 2 sec.)
	**Respiratory rate**	
	0	Normal (66-115 breaths/min)
	1	Abnormal (decreased: < 66 breaths/min or increase: > 115 breaths/min)
	2	Abdominal breathing (**Euthanasia**)
	**Heart rate**	
	0	Normal (250-450 beats/min)
	1	Abnormal (decreased: < 250 beats/min or increased: > 450 beats/min)
	**Body temperature**	
	0	Normal (35.6-38.9°C)
	1	Abnormal (hypothermia: < 35.6ºC or hyperthermia: > 38.9°C)
	**Hematocrit**	
	0	Normal (35-51%)
	1	Abnormal (decreased: < 35% or increased: > 51%)
	**Urine specific gravity**	
	0	Normal (1.040-1.070)
	1	Abnormal (decreased: < 1.040 or increased: > 1.070)
**Mammary tumors**	**Tumors location**	
	0	Do not interfere with animals’ bodily functions
	1	Interfere with animals’ bodily functions (eat or drink) **(Euthanasia)**
	2	Tumor in contact with cage floor **(Euthanasia)**
		**Tumors macroscopic evaluation**
	0	Normal
	1	Erosion
	2	Ulceration (**Euthanasia**, when combined with a size > 35 mm in a 250 g rat**) **
	3	Persistent self-induced trauma **(Euthanasia)**
		**Tumors burden**
	0	< 10% of the animal’s body weight (<35 mm in a 250 g rat)
	1	>10 % of the animal’s body weight (>35 mm in a 250 g rat) (**Euthanasia**, when combined with tumor ulceration)

**Table 2:** Initial and final accurate body weight (g) and ponderal weight gain (%), and mammary tumors’ volume and weight in all experimental groups (mean ± Standard Error)

**Group**	**n**	**Accurate body weight (g)**		**Ponderal weight gain (%)**	**Mammary tumors**	
		**Initial**	**Final**		**Weight (g)**	**Volume (cm** ^3^ **)**
**I (MNU)**	6	188.27 ± 3.78	304.78 ± 8.54	38.10 ± 1.27	3.24 ± 0.66	3.08 ± 0.63
**II (MNU + ketotifen-1)**	8	178.40 ± 3.26	272.78 ± 11.35	33.75 ± 3.09	5.40 ± 1.40	5.11 ± 1.32
**III (MNU + ketotifen-2)**	7	187.69 ± 4.88	286.44 ± 6.72	34.39 ± 1.46	2.62 ± 0.74	2.48 ± 0.70
**IV (ketotifen)**	2	198.22 ± 12.70	304.20 ± 18.00	34.86 ± 0.32	-	-
**V (control)**	2	183.82 ± 2.84	295.98 ± 9.78	37.86 ± 1.10	-	-

Statistically significant differences were not found (*P*>0.05).

**Table 3:** Food and water intake in the first and last weeks of the experimental protocol (mean ± Standard Error).

**Group**	**n**	**Food intake (g)**		**Water intake (mL)**	
		**Initial**	**Final**	**Initial**	**Final**
**I (MNU)**	6	15.90 ± 0.20	20.35 ± 0.59 b	26.09 ± 2.91	36.22 ± 1.62
**II (MNU + ketotifen-1)**	8	15.47 ± 0.17 a	16.15 ± 0.53	23.53 ± 1.52	48.51 ± 8.66
**III (MNU + ketotifen-2)**	7	15.90 ± 0.62	18.76 ± 0.85	25.84 ± 1.43	39.16 ± 17.58
**IV (ketotifen)**	2	17.74 ± 0.00	17.49 ± 0.00	25.90 ± 0.00	47.11 ± 0.00
**V (control)**	2	16.72 ± 0.00	24.02 ± 0.00 c	23.44 ± 0.00	30.18 ± 0.00

a Statistically different from group IV (*P*<0.05);

b Statistically different from group II (*P*<0.05);

c Statistically different from groups I, II, III and IV (*P*<0.05).

***Biological parameters***

No differences were observed in accurate body weight and ponderal weight gain among groups (*P*>0.05) (Table 2). The final food intake was higher in group V (control) when compared with the remaining experimental groups (*P*<0.05) (Table 3). 

In the Table 4 is presented the number of animals from each experimental group that exhibited alterations in any biological parameter evaluated during the study. No changes were observed in animals from groups control (groups IV and V). The first alteration was observed in the 11^th^ week of the experiment in animals from MNU-exposed groups. Only alterations in the body weight, mucosal and tumors burden were observed during the experiment. Slight variations in body weight (score 1, weight loss <10%) were observed from the 11^th ^to the 18^th ^week of the experiment in animals from groups I (MNU) and II (MNU+ketotifen-1). The highest number of animals with alterations in body weight was observed in the 17^th^ week of the experiment, where five animals from group I (MNU) and three animals from group II (MNU+ketotifen-2) exhibited a weight loss <10%. 

**Table 4:** Number of animals from each experimental group (groups I, II and III) with alterations in some of the biological parameters evaluated throughout the experimental protocol

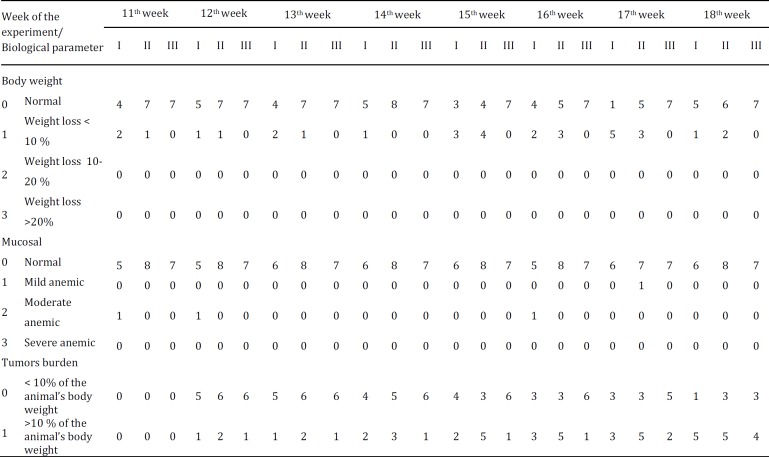

Group I (MNU; n=6), Group II (MNU+ketotifen-1; n=8); Group III (MNU+ketotifen-2; n=7). The first alterations were detected at the 11^th^ week of the experiment. No changes were found in groups IV (ketotifen) and V (control); MNU: N-methyl-N-nitrosourea

Moderate anemic mucosal (score 2) were observed in one animal from group I (MNU) at the 11^th^, 12^th^ and 16^th^ week of the experiment. Only one animal from group II (MNU+ketotifen-1) exhibited alteration of the mucosal (score 1, mild anemic mucosal) at the 17^th ^of the experiment. 

The microhematocrit values were similar among groups and within the physiological values defined by Østergaard* et al.* (33) for rats. The urine from animals from all experimental groups did not show macroscopic alterations. Once the normal values of urine specific gravity for rats range from 1.040 to 1.070 (36), the urine specific gravity was lower than the physiological values in groups I (MNU), II (MNU+ketotifen-1), III (MNU+ketotifen-2) and V (control). Although numerical differences exist among group, they did not reach the level of statistical significance (*P*<0.05).

***Mammary tumors’ development***

The first mammary tumor was simultaneously detected by palpation in all MNU-exposed groups (I, II and III) eight weeks after the MNU administration. Not all MNU-exposed animals developed mammary tumors, an incidence of 60% (6/10) in group I (MNU), 89% (8/9) in group II (MNU+ketotifen-1) and 70% (7/10) in group III (MNU+ketotifen-2) was observed. A total of 58 mammary tumors were counted: 21 tumors in group I, 19 tumors in group II and 18 tumors in group III (*P*>0.05) (Figure 2). No interference of mammary tumors with animals’ bodily functions or alterations in mammary tumor surface that implied animal sacrifice was observed (Figure 3). 

At the 12^th ^week of the experimental protocol, four animals (one animal from group I, two animals from group II and one animal from group III) developed at least one mammary tumor with a diameter >35 mm. After this, the number of animals with at least one mammary tumor >35 mm was increasing and at the end of the experimental protocol 83.3% (5/6) of the animals from group I, 62.5% (5/8) of animals from group II and 57.1% (4/7) of the animals from group III developed at least one mammary tumor >35 mm (Table 4). 

## Discussion

Mammary cancer is the most frequently diagnosed cancer among women worldwide (35). The model of mammary cancer chemically-induced in female rats is one of the most commonly used to study this disease, and to develop new and more effective diagnostic and therapeutic approaches (36).

The establishment of humane endpoints in experimental protocols intend to reduce the severity and/or duration of pain, discomfort and distress experienced by animals (23). Although the concern about the animal welfare has increasing over the years, not only among the researchers but also in the public, the papers reporting the occurrence of distress in animal models of cancer are rare (fifteen scientific papers were found in PubMed when searched the keywords “animal model of cancer” and “welfare”) (37-39).

During the experiments, the animals should be maintained under vigilance and the established systems may be adjusted when necessary (41). Although body condition is considered a simple and noninvasive method for assessing animals’ health and wellbeing (40) and the distress scoring systems for cancer studies are focused on the animal body condition (41), other biological parameters like posture, coat and grooming, mental status, respiratory and heart rate, and body temperature should be considered and evaluated during the experiments. Moreover, the evaluation of more specific indicators of stress and discomfort, like tumor macroscopic appearance and dimensions should be evaluated (42).

When compared with other models of chemically-induced carcinogenesis, the model of mammary cancer has the advantage that the tumors are easily accessible to the researchers, and inversely to that happens with tumor development in other organs such as urinary bladder or liver, they may be measured using a caliper and their macroscopic appearance may be evaluated throughout the experimental protocol (43–45). 

According to the CCAC (28), an appropriate observation of the animals’ condition should not only include changes in body weight, but also related changes in food and water intake. Despite the tumor development, animals MNU-exposed did not exhibit a mean lower body weight nor food and water consumption, when compared with non-exposed animals, suggesting that these animals did not develop cancer-associated cachexia (46, 47). Inversely to that expected for animals with mammary cancer, no changes in biological parameters defined by CCAC (28) like posture, coat and grooming mucosa, eyes, ears and whiskers, mental status, response to external stimuli, hydration status, respiratory and heart rate, and body temperature that implied the premature animal sacrifice were detected during the experiment. 

An incidence of 100% was observed in a previous experimental protocol performed by our research team with the rat model of chemically-induced mammary carcinogenesis (48, 49). However, not all animals exposed to the MNU in the present work developed mammary tumors probably due to the duration of the experimental protocol. The animals from the first experiment were sacrificed 35 weeks after MNU administration while the animals from the present experiment were sacrificed 17 weeks earlier (18 weeks after MNU administration). The first mammary tumor was detected by palpation eight weeks after the MNU administration in all MNU-exposed groups. After this, the animals were developing more tumors and their dimensions were increasing. The first mammary tumor with a diameter >35 mm was detected twelve weeks after MNU administration, and at the end of the experiment 14 animals (5+5+4) from the MNU-exposed groups exhibited at least one mammary tumor >35 mm. Although according to the endpoints established by CCAC, this tumor size is indicator of animal sacrifice (28–30, 32), the animals did not exhibit severe alterations in the remaining parameters and they were only sacrificed at the end of the experiment (18 weeks after MNU administration). 

Additionally to the evaluation of biological parameters and mammary tumors, blood and urine samples were collected and the hematocrit values and urine specific gravity were determined throughout the experiment without significant differences among groups (*P*>0.05). This suggests that these parameters are not valuable for the assessment of the health status of female rats included in an assay of chemically-induced mammary carcinogenesis for 18 weeks. The remaining proposed humane endpoints seemed to be helpful to monitor the animals’ health status.

## Conclusion

Looking to these results, it was possible to conclude that the alteration in only tumor dimensions does not imply the animals’ sacrifice; the endpoints should be evaluated in conjunction, in order to define the most adequate time in which the animals should be humanely sacrificed.
